# Intermetallic and dispersoid structures in AA3104 aluminium alloy during two-step homogenisation

**DOI:** 10.1038/s41598-024-51890-2

**Published:** 2024-02-05

**Authors:** S. L. George, L. Magidi-Chicuba

**Affiliations:** Centre for Materials Engineering, Department of Mechanical Engineering, University of Cape Town, Cape Town, South Africa

**Keywords:** Theory and computation, Mechanical engineering, Metals and alloys

## Abstract

During homogenisation of the AA3104 cast ingot, a phase transformation of intermetallic particles from β-Al_6_(Fe,Mn) orthorhombic phase to harder α-Al_x_(Fe,Mn)_3_Si_2_ cubic phase occurs. The large constituent intermetallic particles, regardless of phase, assist in the recrystallisation nucleation process through particle stimulated nucleation (PSN). Ultimately, this helps to refine grain size. The sub-micron dispersoids act to impede grain boundary migration through a Zener drag mechanism. For this reason, the dispersoids that form during homogenisation are critical to the recrystallisation kinetics during subsequent rolling, with smaller dispersoids being better suited to instances where the minimisation of recrystallisation is required during hot rolling. This work simulates an industrial two-step homogenisation practice with variations in the peak temperature of the first step between 560 °C and 580 °C. The effect of this temperature variation on the intermetallic particle phase evolution is investigated. The aim is to identify the ideal intermetallic phase balance and the dispersoid structure that are best suited for the minimisation of recrystallisation during hot rolling through maximising Zener drag and maintaining galling resistance. The results indicate a trend where an increase in homogenisation temperature from 560 °C to 580 °C yields, firstly, an increase in the volume fraction of the α-phase particles to greater than 50% of the total volume fraction at both the edge and the centre of the ingot and, secondly, it yields an increased dispersoid size. Thus, a lower temperature homogenisation practice produces a near-ideal combination of intermetallic particle phase distribution, as well as dispersoid size, which is critical for Zener drag and the minimization of recrystallisation during the hot rolling processes.

## Introduction

Aluminium alloy AA3104 is currently the most commonly used alloy for the manufacture of the body section of full-aluminium beverage cans. The ends of cans are made from AA5182 alloy, which is an Al–Mg alloy chosen for its strength properties, while AA3104 is selected owing to its formability and recyclability^1^. The production process of the AA3104 can body stock material includes direct chill (DC) casting, hot and cold rolling and coiling and forming. Homogenisation after DC casting is critical for the formation and evolution, with respect to size and density, of critical microstructural features such as intermetallic particles and dispersoids. These features influence both the microstructural evolution and the property development in AA3104 can body stock material during processing and forming into the final product, as well as the galling resistance required for deep drawing and wall ironing during the can manufacturing process.

Intermetallic constituent particles form during solidification of the molten alloy at relatively high temperatures and their sizes lie in the range of one to several tens of microns^2^. The solidification kinetics experienced in DC casting of ingots results in a variation in microstructure and intermetallic structure^3^ between the edge and the centre of the ingot^4^, in terms of grain size^5^, dendrite arm spacing, segregation^6^and intermetallic particle size, phase and distribution.

The relative volume fractions of the intermetallic phases depend on the composition of the alloy and the solidification rate. Faster solidification rates allow the orthorhombic β-Al_6_(Fe,Mn) phase to form in preference to the cubic α-Al(Fe,Mn,Si) phase^3^. The intermetallic particles envelope the dendrites as faceted plate-like phases^7^. There is a greater tendency of the Fe atoms to segregate because the intermetallics formed during solidification contain a higher volume fraction of Fe relative to Mn. There are no ternary-phases that form but, instead, the Mn atoms in Al_6_Mn are substituted by the Fe atoms. During subsequent homogenisation, Mn diffuses more rapidly and enters Al_6_(Fe,Mn) until the phase has achieved maximum solubility^7^.

In order to maximise galling resistance in the AA3104 alloy, the homogenisation process should facilitate the phase transformation of the β-Al_6_(Fe,Mn) to the α-Al_x_(Fe,Mn)_3_Si_x_ intermetallics, with an aim to achieve a 50:50 ratio of the two intermetallic phases. This ratio must occur at both the centre of the ingot and at the edge, where microstructure and segregation levels are different. The 50:50 ratio phase balance is necessary, because the α-Al_x_(Fe,Mn)_3_Si_x_ is a harder phase than the β-Al_6_(Fe,Mn) (800–950 HV vs. 550–740 HV)^5,6,8^. This property increases the galling resistance of the material and reduces seizure during can-forming operations (especially during wall ironing) in materials with a higher alpha to beta ratio^5,8^. However, a too-high volume fraction of the harder α-Al_x_(Fe,Mn)_3_Si_x_ phase may result in an increase in the wear rate of die surfaces. Thus, a balanced volume fraction is desirable.

After ingot solidification the as-cast microstructure contains β-Al_6_(Fe,Mn) as the predominant phase and only traces of α-Al_x_(Fe,Mn)_3_Si_x_ , which can be in the form of α-Al_12_(Fe,Mn)_3_Si or α-Al_15_(Fe,Mn)_3_Si_2_^9^, are seen within the intermetallic particles. Mg_2_Si particles are also present in the microstructure. These particles are critical, as Si diffusion drives the phase transformation when the Si diffuses from the matrix and Mg_2_Si particles into the α-Al_x_(Fe,Mn)_3_Si_x_ phase particles. Therefore, although the eutectoid growth is fast, it is limited by the supply of Si. However, α-phase nucleation is mostly known as the overall rate-controlling factor^10–12^. Furthermore, the microstructure at the centre of the cast ingot shows a lower number of coarser particles compared to the number and size found at the ingot edge^5^. During homogenisation of the cast ingot, diffusion occurs and there is an elimination of micro-segregation, as well as a re-distribution of manganese from solid solution. This diffusion process enables the phase transformation of intermetallic particles from the orthorhombic β-Al_6_(Fe,Mn) phase to the harder α-Al_x_(Fe,Mn)_3_Si_x_ cubic phase on the grain boundaries^1,10^. β-Al_6_(Fe,Mn) undergoes a time- and temperature-dependent eutectoid transformation to α-Al_12_(Fe,Mn)_3_ during homogenisation between 480 °C and 593 °C^5^. The Al formed in the reaction is observed as spots in the alpha matrix that may coalesce at grain boundaries at high temperatures^13^. Initially, α-Al_12_(Fe,Mn)_3_Si nucleates upon heating at the β-Al_6_(Fe,Mn)-Al interface, which is enriched in Si owing to Mg_2_Si dissolution^14^. The alpha phase then grows into the β-Al_6_(Fe,Mn) phase in a solute-dependent process. The transformation is also enhanced by higher homogenisation temperatures, as determined by Alexander et al*.*^13^ and Gandhi^5^. Correspondingly, Goodrich^15,16^ showed that Mn equilibration rates are faster above 550 °C, owing to Mn being more soluble in Al at high temperatures^5,17,18^.

While the large constituent particles, regardless of phase, assist in the recrystallisation nucleation process through particle stimulated nucleation (PSN), the sub-micron dispersoids act to impede grain boundary migration through a Zener drag mechanism^19^. Thus, the dispersoids that form during homogenisation are critical in the recrystallisation kinetics during subsequent rolling. The characteristics of the dispersoids that form during homogenisation vary in size and distribution depending on heat treatment parameters. Smaller dispersoid sizes with high distribution densities retard recrystallisation as a result of Zener drag. Dispersoids are sub-micron particles that primarily consist of α-Al_12_(Fe,Mn)_3_ and Mg_2_Si. α-Al_12_(Fe,Mn)_3_ makes up the majority of the dispersoids after homogenisation^8^. The dispersoids formed during homogenisation become fewer and coarser with an increase in soak temperature^5^. Composition is also critical for dispersoid evolution, where additions of iron and silicon act to reduce the solubility of Mn in the aluminium matrix and accelerate the precipitation of Mn-containing dispersoids. Si favours the precipitation of dispersoids of the simple cubic α-Al_x_(Fe,Mn)_3_Si_x_ phase, while Fe may substitute Mn in both the Al_6_Mn phase and the α-Al_x_(Fe,Mn)_3_Si_x_ phase^20^. During heat treatment, the large β-Al_6_(Mn,Fe) phase begins to fragment and α-Al_x_(Fe,Mn)_3_Si_x_ dispersoids precipitate in dendrite grains^11^. During homogenisation at a temperature of 600 °C, the β-Al_6_(Mn,Fe) dispersoids will form and will have an average size of below 0.1 μm. During a 4-h soaking period, the β-particles coarsen but, at the same time, the total volume fraction of β-particles will decrease slightly. This indicates the dual processes of coarsening and partial re-dissolution of these phases^21^.

This work simulates an industrial two-step homogenisation practice, with variations in the peak soak temperature of the first step. The effect of this temperature variation on the intermetallic particle phase evolution is investigated with the aim to identify the ideal intermetallic phase balance, as well as the dispersoid structures. The intermetallic structures are investigated at both the edge and the centre of the ingot, owing to the inhomogeneous microstructure and particle distribution that result from DC casting. The target ideal intermetallic phase balance is a 50:50 ratio of the orthorhombic β-Al_6_(Fe,Mn) phase to the cubic α-Al_x_(Fe,Mn)_3_Si_x_ phase at both the edge and centre of the ingot.

## Experimental procedure

The AA3104 DC cast sample composition included 0.197 wt.% Si, 0.477 wt.% Fe, 0.197 wt.% Cu, 0.990 wt.% Mn and 1.220 wt.% Mg. Samples were sectioned from locations near the edge and at the centre of a slice taken from the mid-length and mid-width of the cast ingot. The homogenisation profile was based on an industrial homogenisation practice. This two-step industrial homogenisation practice is used to allow for phase transformation of the intermetallic phases, as well as dispersoid structure generation, and both are necessary for recrystallisation control. The benefit of a two-step homogenisation profile is that it results in significantly lower Mn in solid solution, owing to a high degree of dispersoid formation. The second step is a lower temperature in order to avoid dissolution of smaller dispersoids and the coarsening of larger undissolved dispersoids^6^. The homogenisation profiles for this work are divided into two models: Model I and Model II. Model I uses 580 °C as the first step soak temperature for 4 h and Model II uses 560 °C as the first step soak temperature for 4 h. Both Model I and Model II included a subsequent soak at 520 °C for 4 h. The heating and cooling rates were set to 50 °C per hour. Engler et al*.*^22^ identify that most industrial processing of AA3XXX alloys incorporates a single homogenisation at 600 °C or has a multi-step homogenisation in the range of 600 °C. Therefore, the higher temperature of the first soak is in line with industry standards.

The as-cast and homogenised samples were prepared for microstructural image analysis using standard metallographic preparation procedures for aluminium alloys. An etchant of 10% H_3_PO_4_ at 50 °C for 8 min was used to distinguish between the two intermetallic phases within the microstructure, where H_3_PO_4_ reacted with the Si within the alpha particles, turning them dark in colour, while the beta particles remained light grey. The relative phase colouration was confirmed experimentally using a combination of light microscopy and SEM, coupled with EDS. Vander Voort’s relative accuracy equation was used to determine that 200 fields of view (FOV) were necessary to obtain a meaningful particle volume fraction^23^. ImageJ freeware and MATLAB R2013b were used for two-dimensional image analysis in order to estimate the intermetallic particle volume fractions within the etched microstructures, both at the edge and at the centre, before and after homogenisation. A butanol-based matrix dissolution set-up was used to extract intermetallic particles from the bulk samples for three-dimensional volume fraction analysis of the particles. During the butanol dissolution method, the Al matrix is dissolved in dry butanol, leaving only the intermetallic particles behind on a Teflon filter in a purpose-designed autoclave. The first step of the process was to dry 1-Butyl alcohol (Butanol Grade 1–01 AnalaR NORMAPUR® Reag. Ph.Eur., ACS) in an argon (Ar) gas environment at 121 °C. The dry butanol was then transferred into a stainless steel autoclave that was fitted with a 0.1 µm Teflon filter, containing the sample to be dissolved under argon gas. Once the dry butanol had been transferred, the filled pressure vessel was placed in an oven at 135 °C for 8 to 10 h for dissolution to occur. Then, the extracted particles were morphologically and compositionally analysed, using light microscopy and energy dispersive spectroscopy (EDS) in the scanning electron microscope (SEM). Powder x-ray diffraction (XRD) was used for phase identification and quantification. X-ray diffraction, coupled with MDI Jade 5.0 and Topas software, was used to identify the phases present within the particles. The peak intensities are related to the phases that are present. The dispersoid structures are evaluated using bright field transmission electron microscopy (TEM), where 3 mm diameter disks have been extracted, thinned mechanically and prepared using twin-jet polishing. The images were analysed in order to estimate dispersoid particle sizes and distribution.

## Results

### Characterisation of as-cast structure: edge and centre

**Figure 1:**
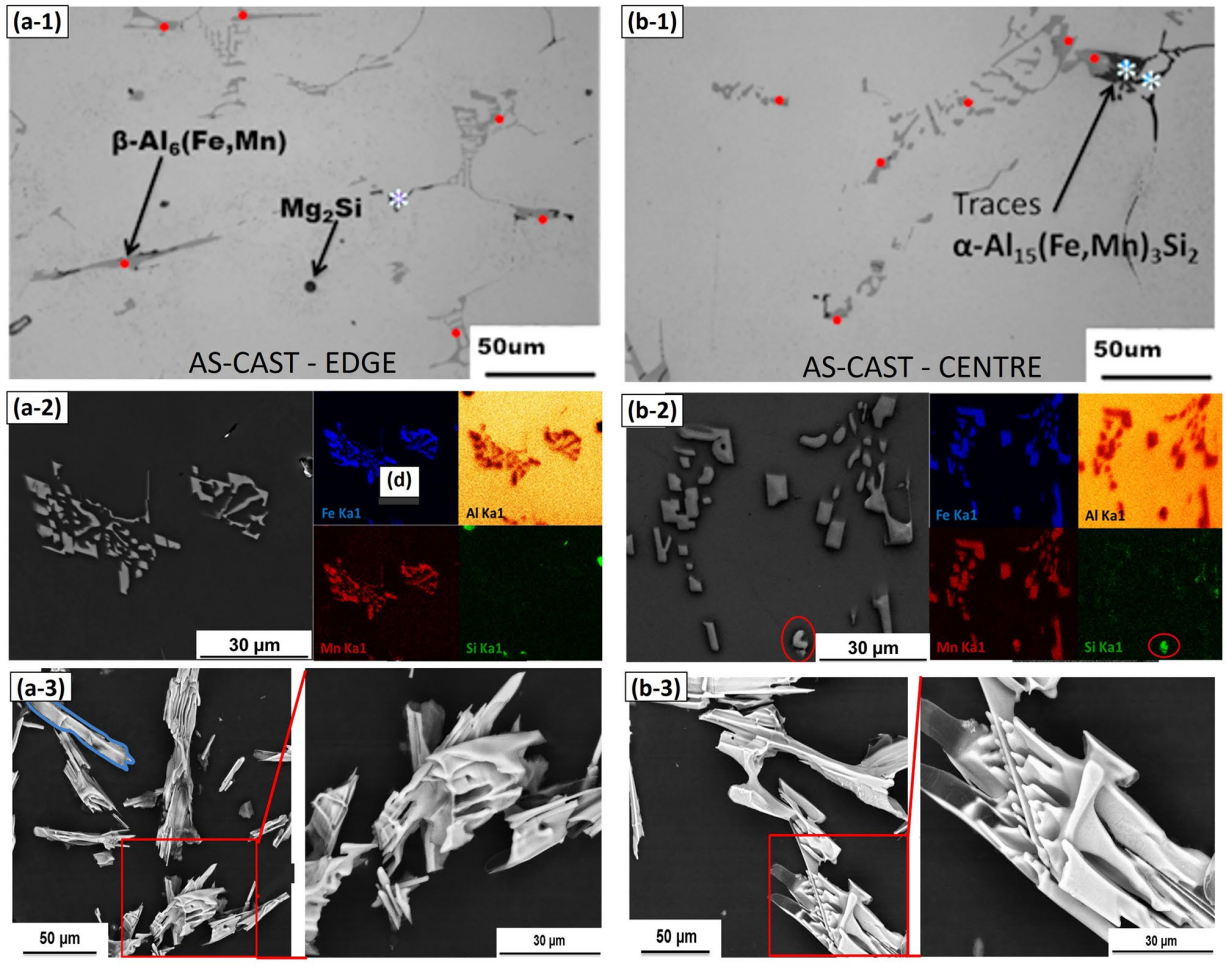
(a-1) to (a-3) show images of the as-cast condition at the edge location, and (b-1) to (b-3) show images of the as cast condition in the centre location. In both cases (1) shows the of β-Al_6_(Fe,Mn) phase indicated with red dots and the α-Al_x_(Fe,Mn)_3_Si_2_ phase indicated with white stars, (2) show SEM Backscattered electron micrographs coupled with EDS maps, where Si-containing α-Al_x_(Fe,Mn)_3_Si_2_ particles are circled in red, and (3) extracted intermetallic particles.

**Figure 2:**
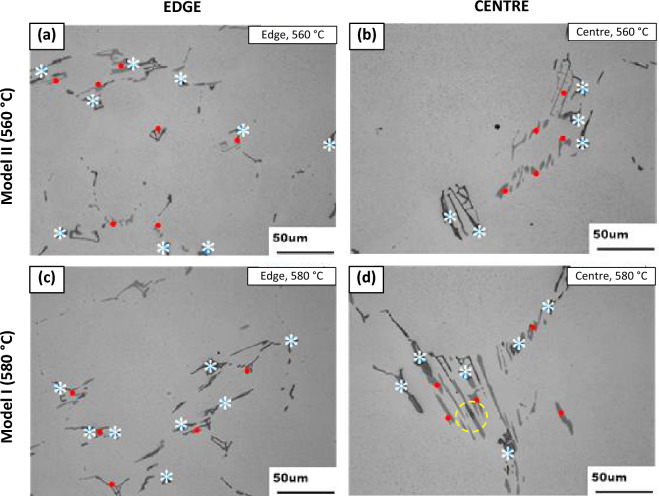
(**a**) & (**b**) show light micrographs of the material after homogenisation at 560 °C, edge and centre respectively and (**c**) & (**d**) show light micrographs of the material after homogenisation at 580 °C, edge and centre respectively. $\beta$-Al_6_(Fe,Mn) phase indicated with red dots and α-Al_x_(Fe,Mn)_3_Si_2_ phase indicated with white stars.

The microstructure of the as-cast AA3104 material was investigated to identify differences between the edge and centre of the ingot, in terms of intermetallic particle morphology, distribution and phase balance. The material was expected to have a total volume fraction of intermetallic particles in the range of 3% of the total volume, which would include a high percentage of the orthorhombic β-Al_6_(Fe,Mn) phase and a lower percentage of the cubic α-Al_x_(Fe,Mn)_3_Si_x_ phase at both edge and centre. Figure 1 shows representative images of the microstructure, taken with light microscopy in (a-1) and (b-1), SEM (BSE imaging) and EDX compositional analysis in (a-2) and (b-2). The results indicated that both the centre and the edge contained a small number of alpha particles, illustrated by the presence of Si in the EDX mappings, and a greater number of beta particles. In Fig. 1a-1,b-1 the alpha particles are indicated with white stars, while the red dots indicate the beta particles. The particles could be differentiated by morphology and greyscale contrast using light microscopy and in BE images in the SEM, with the beta particles having a comb-like and plate-like morphology and the alpha particles having a typical Chinese script morphology. However, elemental contrast was clearer in backscattered imaging in the SEM, as the Z-contrast made the Si-containing α-Al_x_(Fe,Mn)_3_Si_x_ phase appear brighter. This can be seen in the circled particle in Fig. 1b-2.

The as-cast microstructures in Fig. 1 show the presence of both β- and α-particles, as well as Mg_2_Si particles. The predominant phase at this point is β-Al_6_(Fe,Mn) and only traces of the α-Al_x_(Fe,Mn)_3_Si_x_ phase are identified.

**Figure 3:**
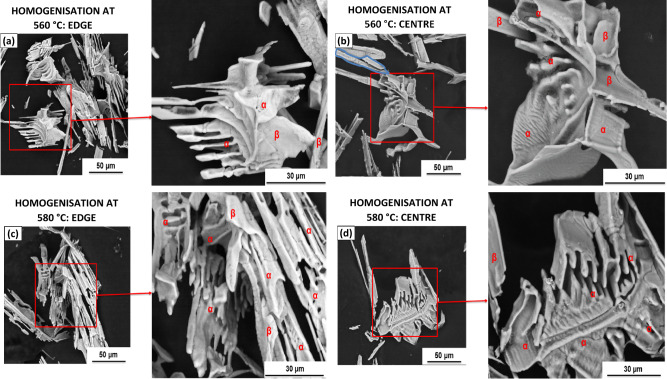
SEM Backscatter electron micrographs showing (**a**) smaller and fragmented comb-like extracted particles with ripples found near the edge of the ingot and (**b**) larger solid extracted particles with ripples found at the centre of the ingot, both after homogenisation at 560 °C, (**c**) fragmented, comb-like and platelet extracted particles found near the edge of the ingot and (d) solid/structured Chinese-script-like extracted particles with ripples found at the centre of the ingot, both after homogenisation at 580 °C. (α and β phases are indicated in the images, as determined using EDS analysis.)

The morphology of the extracted particles can be seen in Fig. 1a-3,b-3 for the as-cast condition. The particles at the edge are smaller in size than those at the centre. The particles in both locations are predominantly β-Al_6_(Fe,Mn), but some α-Al_x_(Fe,Mn)_3_Si_2_ particles are identified. In Fig. 1a-3 the needle-like morphology of an β-Al_6_(Fe,Mn) particle is outlined in blue.

### Characterisation after homogenisation: edge and centre

After homogenisation, the microstructure showed an increase in the relative number of α-phase particles, as seen in Fig. 2. Consistent with the particles in the as-cast condition, the micrographs of the homogenised conditions for both 560 °C and 580 °C showed that the microstructure at the centre contained a lower number of coarser intermetallic particles than at the edge of the ingot. This was explained by the decrease in the solidification rate from the surface to the centre of the ingot, with resultant variations in the microstructural morphology and segregation level. The micrographs also illustrated the phase transformation from β-Al_6_(Fe,Mn) to α-Al_x_(Fe,Mn)_3_Si_2_ that occurred during the homogenisation process for both temperature profiles. The size, morphology and presence of both phases are seen in Fig. 3, which shows images of the 3-D extracted particles. The edge samples appear more fragmented than those at the centre and alpha can be identified from EDX identification in both edge and centre for Model I and Model II conditions.

**Figure 4:**
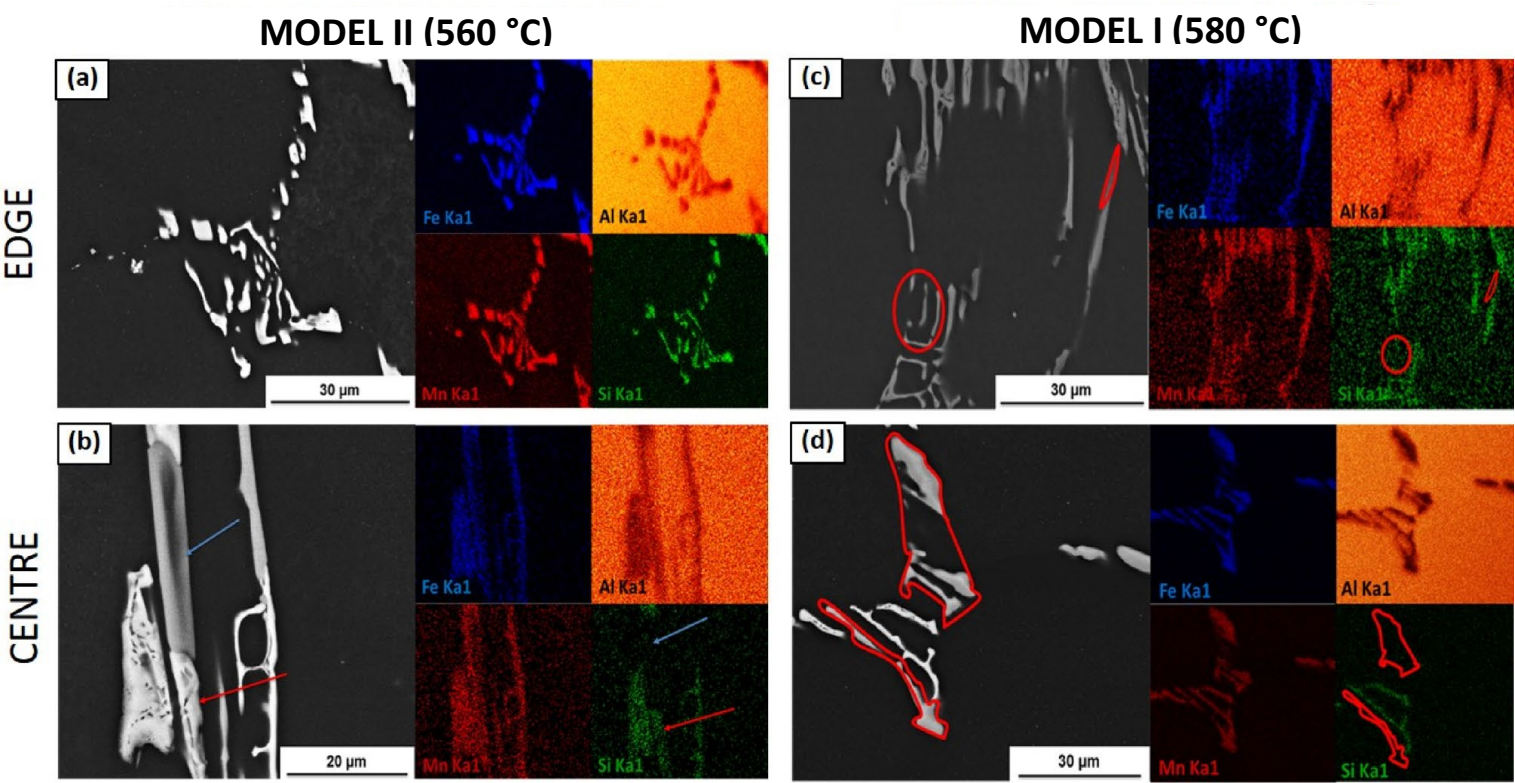
SEM backscatter micrographs, coupled with EDS maps, highlighting the major elements present within the intermetallic particles in Model I and Model II structures. (**a**) shows an α-phase particle in an edge sample and (**b**) a duplex interface with α indicated by the red arrow and β indicated by blue arrow at the centre of the ingot.

The yellow circle in Fig. 2 highlights an example of partial phase transformation as a result of homogenisation at 560 °C, where a partially transformed sample is shown and the junction, or “duplex interface”. The phases were determined using EDS analysis. This same duplex interface was observed by Zhang et al.^24^. They identified a partial phase transformation occurring during the homogenisation of AA3104. Alexander and Greer also observed partial phase transformation^10^. The partial transformation was as a result of Si content, as well as homogenisation time and temperature. For example, a higher Si content or an increased homogenisation temperature or time would result in a higher occurrence of full particle phase transformations. Therefore, if there were a low incidence of partially-transformed particles, it meant that the transformation had occurred at a rapid rate. An example of this is seen in Fig. 4, where homogenisation at 560 °C, as seen in (b), exhibits a clear example of a partial transformation with the presence of a duplex interface, while the equivalent area after homogenisation at 580 °C, as shown in (d), exhibits fully transformed particles, where the the $\beta$-Al_6_(Fe,Mn) phase and the α-Al_x_(Fe,Mn)_3_Si_2_ phase are clearly identifiable by the lack of or presence of Si in the EDS map.

**Figure 5:**
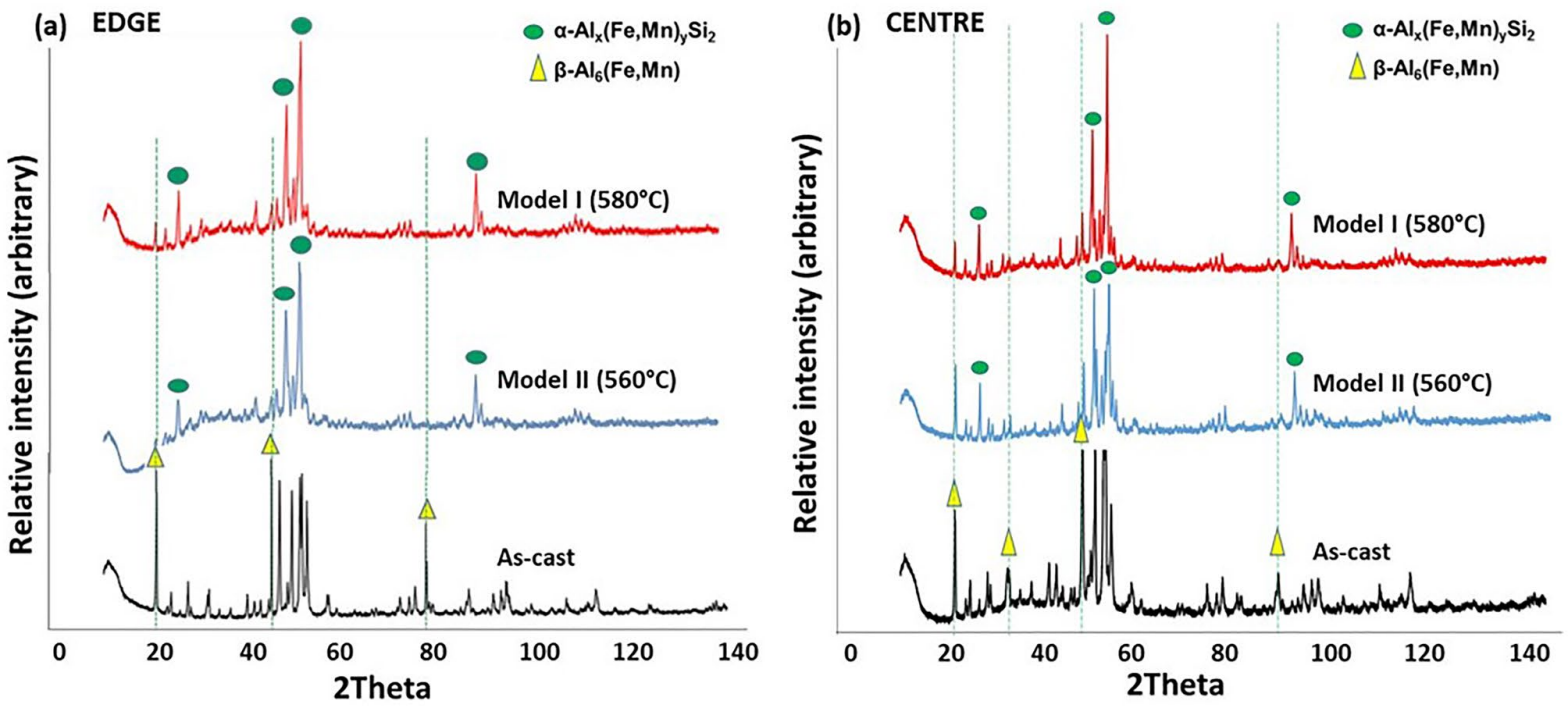
XRD traces showing the phase identification of particles extracted from samples in the as-cast condition, Model I and Model II for (**a**) the **edge** and (**b**) the **centre** of the AA3104 ingot. $\beta$ -phase is indicated by the yellow triangles and α-phase by the green circles as major phases present within extracted particles.

After conducting XRD, a spectrum was generated for each analysed sample in order to identify the phases within the extracted particles. This was achieved by manually indexing the peaks for each specimen, using the crystallographic information of the phase with the MDI Jade 5.0 software. There was no evidence of Mg_2_Si during microstructural characterisation after homogenisation, indicating a full dissolution of the Mg_2_Si particles during the homogenisation process. This was expected at temperatures in this range after short times^25^. Furthermore, based on thermodynamic calculations for this work using thermodynamic modelling software, the following phases were predicted to be present within the AA3104 alloy, with the specified composition and homogenisation temperatures at equilibrium: Mg_2_Si, Al_3_Mg_2_, S-Al_2_CuMg and T-AlCuMgZn. A detailed XRD spectrum with identified peaks for as cast, Model I and Model II samples is shown in Fig. 5. The peaks indexed on the XRD spectrum, not identifiable at the β- and α- peak positions, were assumed to be the other predicted phases in the form of dispersoids. For $\mathrm{\alpha }$-Al_x_(Fe,Mn)_3_Si_2_ the phase major peaks range between 23 and 27, 36–46 and 60–64 degrees^26^, while the $\upbeta$-Al_6_(Fe,Mn) phase major peaks range between 23 and 75 degrees^27,28^.

The dotted lines and yellow triangles in Fig. 5 indicate the strong beta phase peaks present in the as-cast condition. The intensity of these peaks decreases with homogenisation in both Model I and Model II. Peak analysis revealed that the α-phase peaks (green circles) were short and narrow in the as-cast condition but became taller and broader after homogenisation. The peaks also confirmed the presence of $\mathrm{\alpha }$-phase particles in the as-cast condition. There was a distinct decrease in $\upbeta$-phase peaks and an increase in $\mathrm{\alpha }$-phase peaks in Model I and II, showing the progress of the homogenisation-induced phase transformation. The absence of Mg_2_Si in the as-cast condition is not unusual in XRD patterns, owing to the phase being present below the detection limit of the XRD^29^. The as-cast condition showed patterns with high intensity β-phase peaks, while samples in the homogenised condition (Model I and II) showed patterns with α-phase peaks of high intensity and there were a greater number of these α-phase peaks. The decrease in β-phase peaks and the increase in α-phase peaks after homogenisation indicated the extent of the phase transformation that occurred during homogenisation. Further phase peak comparison of the XRD patterns showed that the as-cast samples near the edge contain higher intensity peaks compared to those at the centre. This suggests that samples near the edge contained a greater number of β-phase particles than samples at the centre. Model I and Model II centre samples showed stronger α-phase peaks than did the edge samples, indicating a more dominant β- to α-phase transformation when compared to samples near the edge of the ingot. The phase balance was calculated from XRD using the Rietveld method to estimate the percentage of each phase contained within the volume of extracted particles.

**Figure 6:**
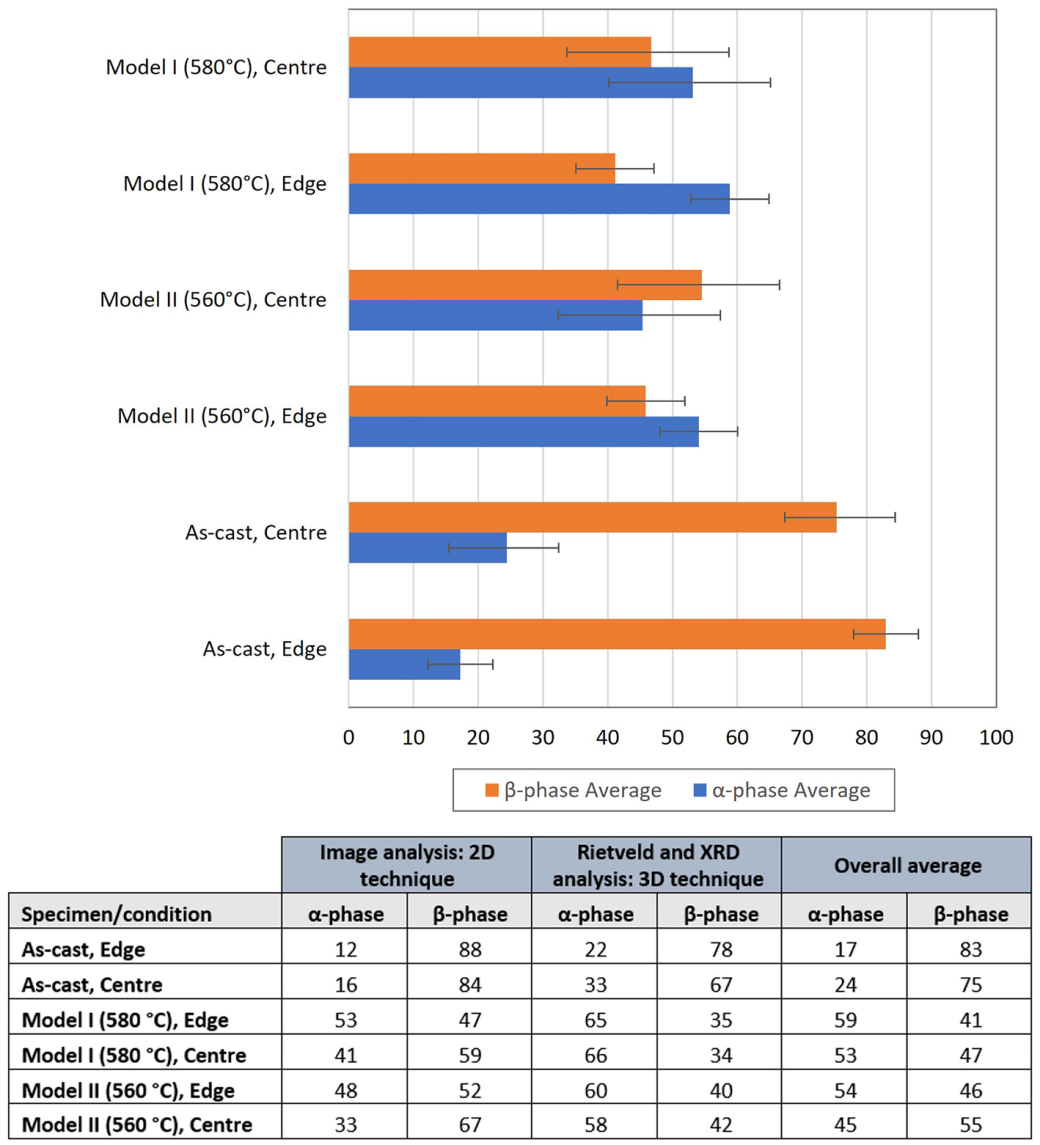
Based on the average of both the image analysis of 2D samples and the Rietveld analysis of 3D powder samples, bar charts of the volume fractions of alpha and beta phases present in the as-cast condition, Model I and Model II, for samples from the edge and centre of the AA3104 ingot. Corresponding calculated values are tabulated.

The volume fraction and phase balance of the β- and α-phases were investigated through two-dimensional analysis of micrographs, while three-dimensional analysis was performed on intermetallic particles extracted from samples exposed to both model I and model II thermal profiles. Neither technique offered a perfect volume fraction value, as each technique would result in interpretation errors but, owing to the statistical approach to the experimental procedure, there was a high level of confidence in the results. A summary of the results for the different conditions, for 2D and 3D analysis is given in Fig. 6. The data are graphically represented and the corresponding calculated values are tabulated, where the overall average volume fraction is given, which is an average of the volume fraction results for both the 2D and 3D techniques.

The results illustrated in Fig. 6 show that, for homogenisation protocol at 580 °C, the α-phase is consistently greater than the critical level of 50% of the total volume fraction of intermetallics. The 50% guideline suggests that a material will offer good galling resistance during deep drawing and wall ironing, without causing excessive die wear. It must be noted that values with higher α-phase volume fractions are associated with die wear and are undesirable. The results indicate that the homogenisation protocol at 580 °C leads to a heterogeneous distribution of α-phase, which would cause excessive die wear.

The 560 °C homogenisation protocol yields $\alpha$ and $\beta$ volume fractions that are close to the desired critical level of 50% of total volume fraction at both edge and centre, but are below 50% at the edge of the ingot. This volume fraction distribution is desirable, as it would offer sufficient galling resistance without resulting in excessive wear^30^.

### Dispersoid characteristics after homogenisation

**Figure 7:**
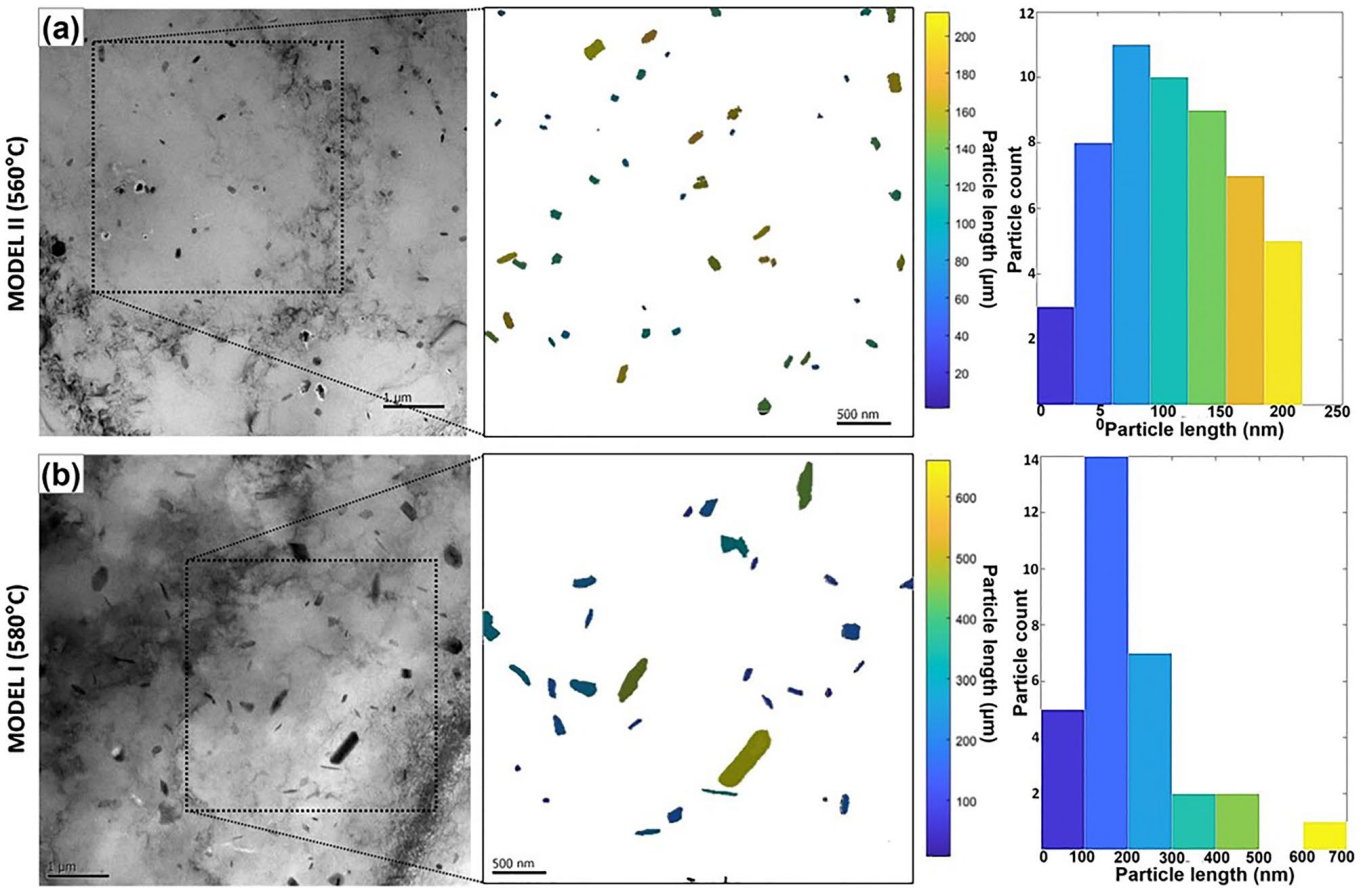
TEM of dispersoid structures for (**a**) Model II (560 °C) and (**b**) Model I (580 °C), with corresponding particle size analysis.

The effect of the homogenisation temperature on dispersoid size and distribution at the centre of the ingot was investigated, as dispersoids are critical for Zener drag. Images were taken of regions from three to five samples for each homogenisation model. The typical size and spatial distributions of the dispersoids within the imaged samples were relatively homogeneous. The representative TEM images in Fig. 7 show the general size and distribution of the dispersoids in the structures of samples that underwent homogenisation at the two different temperatures, namely 560 °C in Fig. 7a and 580 °C in Fig. 7b.

Visual inspection of the TEM images shows that Model II, shown in Fig. 7a, indicates a higher density distribution of smaller dispersoids than those for Model I, shown in Fig. 7b. The typical size range of the dispersoids was determined using image analysis and MIPAR^31^. The dispersoid sizes in Model II ranged between approximately 20 and 200 nm in size, with the average particle size being 114 nm. For Model II, 60% of the total number of particles fell within 47 nm of the average dispersoid size of 114 nm. For Model I, the dispersoids were larger. The typical sizes ranged between approximately 65–470 nm, with the average particle size being approximately 200 nm. For Model I, 60% of the total number of particles fell within 60 nm of the average dispersoid size of 200 nm. Model I, with its higher homogenisation soak temperature, produced a coarser dispersoid structure.

The importance of the dispersoid characteristics relates to the Zener pinning potential of the dispersoids. Where recrystallisation is required to occur later in the process, the presence of smaller dispersoids acts to increase the Zener pinning pressure and reduce recrystallisation^32^. Therefore, recrystallisation and grain growth will be reduced where high Zener pinning pressures are experienced^33,34^, as the Zener pinning pressure and the driving pressure for recrystallisation are opposing forces^35^. Many different models are commonly in use to describe the effect of particle pinning on grain growth behaviour but throughout the models the commonality is the ratio of the particle volume fraction to the particle size^36^. The most simple expression of the Zener drag or pinning pressure can be expressed in the following equation^37^:

$${P}_{z}=\frac{3\gamma {F}_{v}}{2r}$$

where ${F}_{v}$ is the volume fraction of dispersed particles, $r$ is the particle radius and $\gamma$ is the energy of the grain boundary that the dispersed particles are pinning.

$$\frac{{P}_{z}}{\gamma }=\frac{3{F}_{v}}{2r}$$

has been used to calculate a normalised pinning pressure, associated with the two homogenisation models assessed in this work, where the area fraction was calculated from the images and was used as an equivalent value to the volume fraction. The values are shown in Table 1. The results indicate that Model II (560 °C primary soak temperature) had a higher normalised pinning pressure value, which is associated with higher Zener drag and decreased recrystallisation and grain growth.

**Table 1:** Number density of particles, particle radii and area fractions, and normalised Zener pinning pressure values for Model I and II.

	Average particle radius (nm)	Number density of particles per nm^2^	Area fraction of particles	Normalised pinning pressure ($\frac{{{\text{P}}}_{{\text{z}}}}{\upgamma }$) (%)
Model I (580)	79	$1.57\times {10}^{-6}$	0.0313	0.0590
Model II (560)	47	$2.84\times {10}^{-6}$	0.0199	0.0633

### Ethical approval

Ethical approval was attained through the University of Cape Town. No human or animal tissue was used in this work.

## Discussion

The microstructural features, namely intermetallic particles and dispersoids, are critical for microstructural development, mechanical properties and performance in the final product. Intermetallic particle phase balance and distribution are critical for galling resistance in the AA3104 alloy, which is primarily used for beverage can bodies. During forming, these beverage can bodies undergo extensive deep drawing and wall ironing, where the balance between galling resistance and excessive die wear is critical. Any temperature protocol variations in the homogenisation process not only affect the intermetallics, but also influence the formation and coarsening of dispersoid structures within the material, this being critical for microstructure development.

This work focuses on the processing parameters and structure requirements suitable for suppressing recrystallisation during hot rolling. Here, PSN and recrystallisation should be minimised during processing. In addition, the Zener drag contribution of dispersoids is very desirable to retard recrystallisation between passes during rolling and to promote recrystallisation in the post deformation anneal process. Smaller dispersoid sizes with high distribution densities retard recrystallisation, owing to greater pinning pressures relating to increased Zener drag, thus temperature is critical in dispersoid evolution and recrystallisation kinetics later in the rolling process.

The results in this work use two methodologies to gather volume fraction data for the intermetallic particles. The results show that, while the 2D image analysis technique does yield lower volume fractions of α-phase through the thresholding and calculation process across all samples investigated, the relative differences in the edge and centre locations for both Model I and II are consistent with the more complex Butanol-based particle dissolution method. In order to compensate for error resulting from either technique, it was determined that an average of both techniques would yield a better overall value on which to base the results.

The balance between intermetallic particle phase change from the orthorhombic β-Al_6_(Fe,Mn) phase to the harder α-Al_x_(Fe,Mn)_3_Si_x_ cubic phase on the grain boundaries is a diffusion-based process^1,5,6,30^. β-Al_6_(Fe,Mn) undergoes a time- and temperature-dependent eutectoid transformation to α-Al_x_(Fe,Mn)_3_Si_x_, during homogenisation between 480 °C and 593 °C^5^. The temperatures experienced in both of the two-step homogenisation profiles fall within this temperature band, namely Model I (580 °C) and Model II (560 °C) for the first step, both followed by 520 °C for the second step. Initially, α-Al_12_(Fe,Mn)_3_Si nucleates at the β-Al_6_(Fe,Mn): Al interface, which is enriched in Si upon heating, owing to Mg_2_Si dissolution during the homogenisation process^5,14^. The α-Al_x_(Fe,Mn)_3_Si_x_ phase then grows into the β-Al_6_(Fe,Mn) phase in a solute-dependent process, relying on an abundance of Si and Mn in solution at the high temperatures experienced during the first step of homogenisation. The transformation is enhanced by higher homogenisation temperatures, as is shown in the results where Model I produces a high volume fraction of α-Al_x_(Fe,Mn)_3_Si_x_. This is in agreement with the literature^5,13,15–18^.

The 2D analysis results indicate that Model I results in a higher volume fraction of α-Al_x_(Fe,Mn)_3_Si_x_ at the edge of the ingot compared to the centre, and the 3D analysis results indicate that there is little difference between the edge and the centre when ratios of extracted particles per unit volume are used. But in both cases, the volume fractions for the harder α-Al_x_(Fe,Mn)_3_Si_x_ are greater than the desired 50:50 ratio of β- to α-phase. The unfavourable α-phase values and variability of the intermetallic particle phase balance between edge and centre, owing to the higher temperature homogenisation, are compounded by the coarser dispersoid structures, with an average dispersoid size of 200 nm, as opposed to the 114 nm size particles resulting from Model II.

The results indicate that Model II, where the primary soak temperature is 560 °C, produces a structure with a higher distribution of smaller dispersoids, resulting in an increase normalised Zener pinning pressure. Thus, this results in the retardation of the onset of recrystallisation. The higher pinning pressure will directly affect the recrystallisation and grain growth when based on the methodologies and results presented by Eivani et al.^37^. The microstructural features that result are smaller dispersoid sizes with an average equivalent radius of 47 nm with high distribution densities. These features retard recrystallisation because of Zener drag, thereby allowing for a delay in the recrystallisation of the microstructure to later in the process stream during a post deformation batch anneal. This dispersoid structure created in Model II, combined with the relatively even distribution of both the α-Al_x_(Fe,Mn)_3_Si_x_ and β-Al_6_(Fe,Mn) phase intermetallic particles, at a phase ratio of 46:54 (β- to α- phase) at the edge and 55:45 at the centre, is favourable. These ratios are close to the desired 50:50 ratio at the edge and centre, which is suitable for galling resistance.

## Conclusions

In the context of multiple reductions on a hot finishing mill, Model II indicates the development of favourable microstructural features. Model II, which incorporates a lower temperature in the primary step of this specific two-step industrial homogenisation practice of 560 °C for 4 h, yields a desired combination between intermetallic particle phase balance and distribution, as well as smaller dispersoid structures within the AA3104 ingot material. Model I, where the primary step soak temperature is higher (580 °C), produces a greater than 50% volume fraction of the harder cubic α-Al_x_(Fe,Mn)_3_Si_x_ phase within the intermetallic particles at both the edge and the centre of the ingot. The calculated volume fraction at the edge is 59%, which is undesirable for the can body makers, as the material will result in wear of the dies during deep drawing and wall ironing. The primary disadvantage of Model I is the large dispersoid sizes that are produced, which negatively affect the Zener-drag requirements. Thus, Model II, with the primary step soak temperature of 560 °C, is better suited to multiple stage hot rolling, as well as meeting forming property requirements for manufacture.

## Data Availability

The data that support the findings of this study were used under license for the current study, and so are not publicly available. Data are however available from the corresponding author (sarah.george@uct.ac.za) upon reasonable request and with permission of Hulamin Rolled Product. The authors have permission to publish any and all proprietary information contained in this article.
